# MIXED-METHOD PILOT STUDY OF BREAST AND PROSTATE CANCER TREATMENT DIFFERENCES BY COGNITIVE STATUS

**DOI:** 10.1093/geroni/igad104.0090

**Published:** 2023-12-21

**Authors:** Megan Mullins, Mohammed Kabeto, Paul Abrahamse, Sarah Hawley, Deborah Levine, Lindsay Kobayashi, Lauren Wallner

**Affiliations:** UT Southwestern Medical Center, Dallas, Texas, United States; University of Michigan, Ann Arbor, Michigan, United States; University of Michigan, Ann Arbor, Michigan, United States; University of Michigan, Ann Arbor, Michigan, United States; University of Michigan, Ann Arbor, Michigan, United States; University of Michigan School of Public Health, Ann Arbor, Michigan, United States; University of Michigan, Ann Arbor, Michigan, United States

## Abstract

We evaluated the association of cognitive status with treatment for breast and prostate cancer in the US Health and Retirement Study (HRS) linked to Medicare claims (2000-2016), and surveyed physicians at an NCI designated comprehensive cancer center (N= 20, response rate 52%) to explore whether treatment recommendations differ by patient cognitive status. We evaluated the association between cognitive status and treatment receipt among Medicare-eligible HRS participants aged ≥65 whose first and only cancer was breast (n=334) or prostate (n=1,210). Multivariable logistic regression was used adjusting for race, marital status, year, age, activities of daily living, instrumental activities of daily living, education, and comorbid conditions. Physicians were randomized to clinical vignettes presenting a patient with mild cognitive impairment (MCI) or normal cognition and asked their treatment recommendations for early-stage cancer via survey. Women with dementia had 80% lower odds of receiving radiation therapy compared to cognitively normal women (aOR 0.17; 95% CI: 0.03-0.87). Oncologists were less likely to recommend chemotherapy for early-stage breast cancer patients with MCI (vs. normal cognition). Prostate cancer treatment receipt and recommendations did not vary by cognitive status. Notably, 25% of breast oncologists and 40% of urologic oncologists overestimated the risk of dementia among patients with MCI, and both groups were more likely to ascertain caregiver preferences for patients with MCI. These findings suggest there may be differences in cancer treatment recommendations and receipt among patients with MCI compared to those with normal cognition, which may be in part driven by physician overestimation of dementia risk.

